# Parent proxy assessment of sibling quality of life following pediatric hematopoietic cell transplantation

**DOI:** 10.1186/s12955-019-1231-9

**Published:** 2019-10-29

**Authors:** David Buchbinder, Sunita K. Patel, Jacqueline N. Casillas, Diane J. Nugent, Steven Neudorf, Leonard S. Sender, Lilibeth Torno, Heather Huszti, Lonnie K. Zeltzer, Susan K. Parsons

**Affiliations:** 1 Department of Pediatrics and Division of Pediatric Hematology, CHOC Children’s Hospital and University of California at Irvine’s Chao Family Comprehensive Cancer Center, Irvine, California USA; 2 CHOC Children’s Hospital, 1201 W. La Veta Avenue, Orange, CA 92868 USA; 3 Department of Cancer Control and Population Sciences, City of Hope, Duarte, California USA; 4 Department of Pediatrics and Division of Pediatric Hematology and Oncology, David Geffen School of Medicine at University of California at Los Angeles and University of California at Los Angeles’s Jonsson Comprehensive Cancer Center, Los Angeles, California USA; 5 Department of Pediatrics and Division of Hematology at CHOC Children’s Hospital, Orange, California USA; 6 Department of Pediatrics and Division of Oncology at CHOC Children’s Hospital, Orange, California USA; 7 Department of Pediatrics and Division of Psychology at CHOC Children’s Hospital, Orange, California USA; 8 Psychiatry / Biobehavioral Science, and Division of Cancer Prevention and Control Research, David Geffen School of Medicine at University of California at Los Angeles and University of California at Los Angeles’s Jonsson Comprehensive Cancer Center, Los Angeles, California USA; 9 Institute of Clinical Research and Health Policy Studies at Tufts Medical Center and Departments of Medicine and Pediatrics, at Tufts University School of Medicine, Boston, Massachusetts USA

**Keywords:** Family, Quality of life, Hematopoietic stem cell transplantation, Sibling

## Abstract

**Background:**

When a child undergoes hematopoietic cell transplantation (HCT), the impact extends to the entire family, including siblings. Assessment of the quality of life (QoL) of siblings is challenged by their general lack of availability for regular assessment by clinical providers. Thus, the use of parent proxy reporting may be useful. Our aim was to describe the QoL of siblings of HCT survivors, as reported by their parents, as well as to identify parent and family factors associated with lower sibling QoL.

**Methods:**

A cross-sectional study was utilized to assess parent-reported QoL of the HCT recipient’s sibling (Short Form (SF)-10 Health Survey for Children and the Pediatric Symptom Checklist (PSC)-17). Parent QoL was assessed using the SF-12. Multivariable linear regression was used to explore hypothesized predictors of sibling QoL, including parent QoL, family impact/function (Impact on Family Scale, Family Adaptability and Cohesion Evaluation Scales, IV, and a question asking about financial problems) while adjusting for demographic and HCT characteristics.

**Results:**

Ninety-seven siblings (55% males) with a mean age of 12 years (standard deviation [SD] 4 years) were assessed, representing HCT survivors, who were an average of 5 years (SD 4 years) post-HCT. Neither sibling psychosocial (mean 49.84, SD 10.70, *p* = 0.87) nor physical health scores (mean 51.54, SD 8.42, *p* = 0.08) differed from norms. Parent proxies reported behavioral/emotional problems (PSC-17 total score > 15) in 24% of siblings. While parental ratings of their own physical health (SF-12 were higher than norms (mean 53.04, SD 8.17, *p* = 0.0005), mental health scores were lower (mean 45.48, SD 10.45, *p* < 0.0001). In multivariable analysis, lower parent emotional functioning and adverse family function were associated with lower sibling QoL, as reported by parents.

**Conclusions:**

While proxy-reported QoL of siblings did not differ significantly from normative data, both parent QoL and family function were associated with sibling QoL. Future research is needed to understand how siblings themselves perceive their QoL following HCT.

## Background

Hematopoietic cell transplant (HCT) is utilized to treat a variety of malignant and non-malignant disorders [1]. High doses of chemotherapy often in combination with irradiation are used to ablate hematopoietic stem cells as well as cells of the immune system which mediate rejection. This is followed by the infusion of healthy donor hematopoietic stem cells derived from various stem cell sources such as bone marrow. Improvements in HCT supportive care and the use of less toxic approaches have led to a growing cohort of HCT survivors [2]. By 2030, it is estimated that there will be 0.5 million HCT survivors in the United States (US) [3]. As average US families have two children, [4] this also translates to a growing population of siblings of survivors of HCT. The NCI’s Office of Cancer Survivorship notes that the survivorship experience impacts all members of the family, including the patient’s siblings. This family impact may play a critical role in the survivor’s ability to adapt and progress towards positive outcomes [5].

Despite its importance, there is limited research about the impact of the HCT experience on siblings’ health and overall well-being [6–9]. As defined by the World Health Organization, health is a state of physical, mental, and social well-being [10]. For the purposes of this study, utilizing the framework of Wilson and Cleary, we acknowledge the complexity of the construct of QoL and related terms such as health status [11]. These terms are often used interchangeably; however, for our purposes QoL refers to the well-being of the sibling with respect to various aspects of health and well-being including physical, mental, and social health [11]. The ability to assess QoL of siblings of pediatric HCT survivors is challenged by a variety of factors including their absence in clinic and at the bedside. While sibling self-report is preferred and should be considered, the circumstances surrounding the sibling HCT experience underscores the need to characterize and understand the role of proxy reporting in the assessment of QoL among siblings of HCT survivors. Therefore, it is vital to characterize and better understand the role of parent proxy assessment of sibling health status following HCT.

### Siblings’ QoL following transplant

HCT may be perceived and experienced as highly stressful by all children in the family. In a study of 44 siblings of HCT survivors, one-third demonstrated a moderate to severe posttraumatic stress reaction [6]. A study of 33 siblings of 2-year HCT survivors found that parent proxy reports of internalizing emotional and behavioral problems, as measured by the Child Behavior Checklist, were greater among siblings when compared to HCT survivors. In contrast, the groups were not different on QoL [7]. Interviews with siblings, as well as with parents about the siblings, further document a spectrum of internalizing and externalizing emotional disturbances and associated behavioral changes. These emotional and behavioral difficulties were observed both prior to and during HCT [8, 9]. Positive changes have also been documented [8, 9]. Medical characteristics, such as donor status, have been investigated as another source that might influence psychological adjustment in siblings of children undergoing HCT [6]. The survival status of the HCT recipient also influences the siblings’ emotional reactions [8].

### Parent QoL following transplant

Parents of children undergoing HCT experience stress, which is associated with lower parent QoL. A study of 111 mothers of HCT recipients at 18 months post-HCT by Manne et al., documented that 20% demonstrate symptoms consistent with a diagnosis of major depressive disorder, generalized anxiety disorder, panic disorder, or post-traumatic stress disorder (PTSD) [12]. The presence of these disorders at the time of transplant was associated with lower parental QoL post-HCT. In a study of 73 parents of HCT survivors, parental stress even 5 years after the child’s HCT was elevated at similar levels to a comparison population of parents of children newly diagnosed with cancer [13].

### Family impact and function following transplant

The HCT experience may result in family disruption which may be associated with the development of lower QoL among siblings. The disruption of the family can be related to geographic separation of the sibling from the family. Changes in employment status or school status may also lead to family disruption. In a study of 64 families, pre-HCT family environment variables were predictive of HCT survivor adjustment post-HCT [14]. Family functioning is also a predictor of parent adjustment as documented in a study of 151 parents in which domains of family cohesion and conflict were predictors of parent distress [15]. Family functioning has been associated with sibling psychosocial adaptation in the context of the childhood cancer experience [16].

### Transplant-related factors

Survivors’ HCT-related characteristics may serve as risk factors for the development of adverse sibling QoL. Although focused on parent distress, a recent study of 165 HCT recipients during the first year post-HCT documented that the presence of early complications and late complications such as severe infection was associated with diminished parental emotional functioning [17]. Having a brother or sister who survived HCT, but who was experiencing complications and associated poor health, mentally and/or physically, may put more chronic strain on the parents, but may also decrease the amount of support and attention the sibling receives. Such a situation may strain the survivor-sibling relationship and serve as a reminder of the HCT experience.

### Aims of present study

The aim of this study was to describe the QoL of siblings of pediatric HCT survivors, as reported by their parents, and to identify factors associated with lower (worse) QoL outcomes among the siblings. Specifically, we addressed the following questions:

1. Do siblings of HCT survivors have lower (worse) QoL than population norms?
2. Do parents themselves have lower (worse) QoL than population norms and is this associated with lower (worse) sibling QoL?
3. What is the association between family impact and function and sibling QoL?

We hypothesized that siblings of HCT survivors are at risk for lower QoL, as reported by their parents, and that their parents are also at risk for lower self-reported QoL. We expected that adverse parent QoL, family impact and problematic family functioning (less cohesion, greater disengagement, greater enmeshment, less flexibility, greater chaos, greater rigidity) would be associated with lower (worse) sibling QoL as reported by parents adjusting for selected demographic and HCT-related factors (e.g., malignant diagnosis, allogeneic HCT, etc.).

## Methods

### Study design and recruitment

The study employed a cross-sectional study design and recruited participant families from three sites in the Los Angeles and Orange County areas, including CHOC Children’s Hospital, Mattel Children’s Hospital at UCLA, and City of Hope Medical Center. Inclusion criteria included the following: 1) parents of siblings (ages 5–17.9 years) of HCT survivors, 2) at least 1-year post HCT and 3) parents that were English or Spanish speaking. Exclusion criteria included the following: 1) parents of siblings (ages < 5 years or > 18 years), 2) less than 1-year post HCT and 3) parents that were not English or Spanish speaking. Only eligible parent participants were invited to participate in the study. One parent from each individual family was asked to consider one donor or one non-donor sibling (closest in age to the survivor) from their family to avoid over-representation from individual families. The study was approved by the CHOC Children’s Hospital Institutional Review Board (IRB) (IRB #110660) and at all collaborating centers. In addition to the documentation of informed consent, parent participants signed a release granting access to the HCT survivor’s medical records. Diagnostic and HCT-related information was obtained from the survivors’ treating institution by medical record abstraction.

Eligible families were invited to participate in this study from November 2011 through April 2014 at CHOC Children’s Hospital (coordinating site), Mattel Children’s Hospital at UCLA, and City of Hope Medical Center. Recruitment occurred over a prolonged period to account for lower HCT center volume at the coordinating site than anticipated, necessitating the addition of two recruitment sites. A total of *N* = 93 participant families recruited provided completed questionnaires. Overall response rates were available from the coordinating site (CHOC) in which 66 families were eligible. Four families declined for various reasons (not interested, no time, not an appropriate family, eye irritation) giving an overall response rate of 94%. Similar information was not collected by other recruiting sites.

### Measures

#### SF-10 health survey for children

Sibling QoL served as the primary outcome measure for the study and was measured using the Short Form (SF)-10 Health Survey for Children [18]. The SF-10 is a parent-completed measure of 10 questions derived from the full version of the Child Health Questionnaire, which was chosen for its ability to measure physical, mental, and social aspects of a child’s health and well-being. The SF-10 items measure eight domains of health including physical functioning, role limitations due to physical health, bodily pain, general health perceptions, vitality, social functioning, role limitations due to emotional problems, and mental health. The SF-10 is scored to produce a physical health summary (PHS) score and psychosocial health summary (PSS) score. The PHS and PSS scores are converted to T-scores using age and sex-specific community normative data, which have a mean of 50 and standard deviation of 10. Higher T-scores correspond to greater/better QoL. The SF-10 has demonstrated acceptable psychometric properties [19, 20].

#### Pediatric symptom checklist 17

Additional aspects of sibling QoL were measured using the Pediatric Symptom Checklist (PSC)-17 [21]. The PSC-17 is a screening measure, designed to facilitate the recognition of mental health problems in children. A parent-completed version of the PSC-17 was utilized which consisted of 17 items that are rated as “Never,” “Sometimes,” or “Often” present and scored 0, 1, and 2, respectively. A total problem score ranging from 0 to 34 was generated. The total problem score was dichotomized, where clinically “elevated” represents a total score > 15. The PSC-17 has demonstrated acceptable psychometric properties [22, 23].

#### SF-12

Parent QoL was assessed using the SF-12 [24]. The SF-12 items assess domains of physical functioning, role functioning physical, bodily pain, general health, vitality, social functioning, role functioning emotional, and mental health. Composite scores including a physical component summary (PCS) score and a mental component summary (MCS) score were calculated. The SF-12 scores are converted to T-scores using age and sex-specific community normative data, which have a mean of 50 and standard deviation of 10. Higher T-scores correspond to greater QoL. The SF-12 has demonstrated acceptable psychometric properties [25, 26].

#### Impact on family scale

Perceived impact of HCT on the family was assessed using the Impact on Family (IOF) Scale [27]. This impact includes aspects such as the financial burden, emotional concerns of family members as well as positive outcomes associated with illness. The IOF consists of 15 items reflecting the impact of chronic childhood illness on social and family functioning. Items are rated as “Strongly Agree”, “Agree”, “Disagree”, “Strongly Disagree” and scored to compute a total impact score. Higher scores reflect adverse impact of the child’s condition on the family. The IOF has demonstrated acceptable psychometric properties [28, 29].

#### Family adaptability and cohesion evaluation scales

Family functioning was assessed using the Family Adaptability and Cohesion Evaluation Scales, Version IV (FACES IV) [30]. FACES IV measures specific aspects of family functioning including family cohesion and family flexibility. The FACES IV consist of 42 items reflecting the impact of chronic childhood illness on family functioning across two dimensions including flexibility and cohesion. Items are rated as “Strongly Agree”, “Generally Agree”, “Undecided”, “Generally Disagree”, “Strongly Disagree” and scored to compute scores for six scales, each comprised of 7 items. Two balanced scales (cohesion, flexibility) as well as four unbalanced scales which represent the extremes of cohesion and flexibility (disengaged, enmeshed, rigid, and chaotic) are represented. Higher scores on the balanced and unbalanced scales represent adaptive and maladaptive family function; respectively. FACES IV has demonstrated acceptable psychometric properties [31, 32].

#### Sociodemographic and transplant-related factors

Sociodemographic information was self-reported by parent respondents including survivor age, gender, educational attainment (dichotomized as high school education or greater versus other), parent sex, age, number of children living at home, marital status (dichotomized as married versus other), race / ethnicity, annual household income (dichotomized as >$40,000 versus $40,000), health insurance status (dichotomized as insured versus other). A single question also explored if the health condition resulted in financial problems for the family, derived from the National Survey on Children with Special Healthcare Needs [33]. HCT-related information included age of HCT survivor, time since HCT, type of HCT (autologous versus other), HLA compatibility (matched versus other), stem cell source (marrow versus other), conditioning regimen (total body irradiation (TBI)-based versus none and busulfan-based versus none), diagnosis (non-malignant versus malignant), length of hospitalization, disease recurrence following HCT, and the occurrence of acute and chronic graft-versus-host disease (GVHD), as documented in the medical record.

Among the validated questionnaires, the English and Spanish versions were used. For study-specific measures (e.g., the sociodemographic information questionnaire) unavailable in Spanish, the English version was translated and then back translated by a certified professional translator with bilingual proficiency in both English and Spanish.

### Analysis

Mean summary scores (SD) were calculated from established scoring algorithms for all study measures. For sibling QoL, comparisons were made with population norms using a one-sample T-test (null hypothesis of the sample mean = 50), as well as calculating the percentage of children whose QoL was described as low (e.g., one standard deviation below the T-score mean of 50). The proportion of parent respondents endorsing a clinically “elevated” total problem score (> 15) on the PSC-17 was calculated. For parent QoL, SF-12 composite scores were compared to population norms using a one-sample T-test (null hypothesis of the sample mean = 50). Descriptive data (mean scores and standard deviations) for family impact (IOF scores, FACES IV scores), sociodemographic and HCT variables were summarized.

Univariable linear regression was utilized to explore associations between sibling QoL (i.e., SF-10 T-scores), sibling adjustment scores (i.e., PSC-17 scores) and factors including: parent QoL (i.e., SF-12 scores), family impact/function (i.e., IOF scores, FACES IV scores), sociodemographic, and HCT-related variables. Any parent QoL, family impact/function, sociodemographic and HCT-related predictor variables that were significant at a *p* ≤ 0.10 level were included in the multivariable modeling. For the primary outcome (SF-10) and the secondary outcome (PSC-17 scores), parent QoL scores (SF-12) as well as family impact and function scores (IOF, FACES IV), were entered into the multivariable model adjusting for the relevant sociodemographic and HCT-related variables. All tests of significance were two-sided and statistical significance was defined as *p* < 0.05. SAS version 9.3 (Cary, NC) was utilized for all analyses.

## Results

Descriptive characteristics of study respondents, siblings, and HCT-related characteristics are presented in Table 1. The mean age of parent participants was 40 years (SD, 8.7). Most of study respondents were female (84%), married or living with a partner (81%), and self-identified as Hispanic /Latino (59%). Approximately half of the same reported an average household income of less than $40,000. Most study respondents had health insurance for their HCT survivor (97%).

**Table 1:** Demographics of the study population (*n* = 97)

Variable	Value
Sibling Characteristics	
Mean Age (standard deviation)	11.8 years (4.2)
Male (%)	54.7
Mean number of siblings (standard deviation)	2.1 (1.1)
Parent Characteristics	
Mean Age (standard deviation)	39.7 years (9.2)
Male (%)	15.5
No. Latino (%)	59.4
No. Married (%)	81.4
No. Income >$40,000 (%)	50.5
No. Insured (%)	96.8
No. Financial Problems (%)	61.5
Transplant Characteristics	
Mean time since transplant (standard deviation)	4.9 years (3.7)
No. Non-malignant diagnosis (%)	28.9
No. Auto transplant (%)	16.5
No. HLA matched (%)	74.7
No. Marrow (%)	69.1
No. total body irradiation based (%)	33
No. busulfan based (%)	39.4
No. recurrence (%)	10.3
No. acute graft-versus-host disease (%)	56.4
No. chronic graft-versus-host disease (%)	40.5

The sample of siblings that parents reported on had a mean age of 12 years (SD, 4.5). Most of the siblings were male (55%). Approximately one-third of siblings were donor siblings. The average number of siblings was two in respondent families. The mean time that had elapsed since HCT was 5 years (SD, 3.7). The majority of HCT procedures were conducted for treatment of a malignant disorder (71%). A greater proportion of survivors had received an allogeneic HCT (83%), using marrow (69%) as a graft source. Conditioning exposures included TBI and busulfan, 33 and 39%; respectively. Acute GVHD occurred in 56% of cases and chronic GVHD occurred in 40% of cases.

### Sibling QoL

Parent proxy assessments of sibling psychosocial health represented by mean SF-10 PSS scores 49.84 (SD 10.70) for the sample was not different from normative data (one sample t-test, *p* = 0.87). Similarly, parent proxy assessments of sibling physical health represented by PHS mean scores (mean 49.84, SD 10.70) was not different from normative data (one sample t-test, *p* = 0.08). However, a total of 17.8% of parents noted that siblings had psychosocial health represented by PSS scores were one standard deviation below the normative mean, whereas a total of 9.2% of parents noted that siblings had physical health represented by PHS scores that were one standard deviation below the mean. Parent proxies reported behavioral/emotional problems (PSC-17 total score > 15) in 24% of siblings.

### Parent QoL

Parents’ SF-12 scores differed from normative data. While parent physical health represented by mean PCS scores of 53.04 (SD 8.17) were higher than normative data (one-sample t-test, *p* = 0.0005), parent mental health represented by mean MCS scores of 45.48 (SD 10.45) were lower than normative data (one sample t-test, *p* < 0.0001). A total of 7.2% of parents reported physical health represented by PCS scores that were 1 SD below the mean. A total of 28.6% of parents reported mental health represented by MCS scores that were 1 SD below the mean.

### Family impact and functioning

Family impact was represented by mean total IOF scores of 35.86 (SD 8.90). Family functioning was represented by FACES IV mean subscale scores of cohesion 27.85 (SD 4.82), flexibility 26.07 (SD 4.54), disengaged 15.07 (SD 5.16), enmeshed 16.36 (SD 4.99), rigid 21.26 (SD 4.30), and chaotic 14.87 (SD 5.15).

### Factors associated with lower (worse) sibling QoL

The results of univariable linear regression analyses, which examined factors associated with sibling QoL as reported by parents, are displayed (see Tables 2 and 3). Tables 4, 5 and 6 depict the results of multivariable linear regression models describing aspects of parent QoL, family impact and family function associated with parent proxy-reported sibling QoL controlling for relevant sociodemographic and HCT-related factors. Lower parent QoL including mental health was associated with lower (worse) sibling psychosocial health. Lower parent QoL including physical and mental health was associated with a greater burden of emotional / behavioral problems as reported by parents. Family functioning and more specifically diminished family cohesion was associated with lower (worse) sibling psychosocial health as reported by parents. Diminished household income was associated with lower (worse) sibling psychosocial health as reported by parents although this achieved only marginal statistical significance.

**Table 2:**
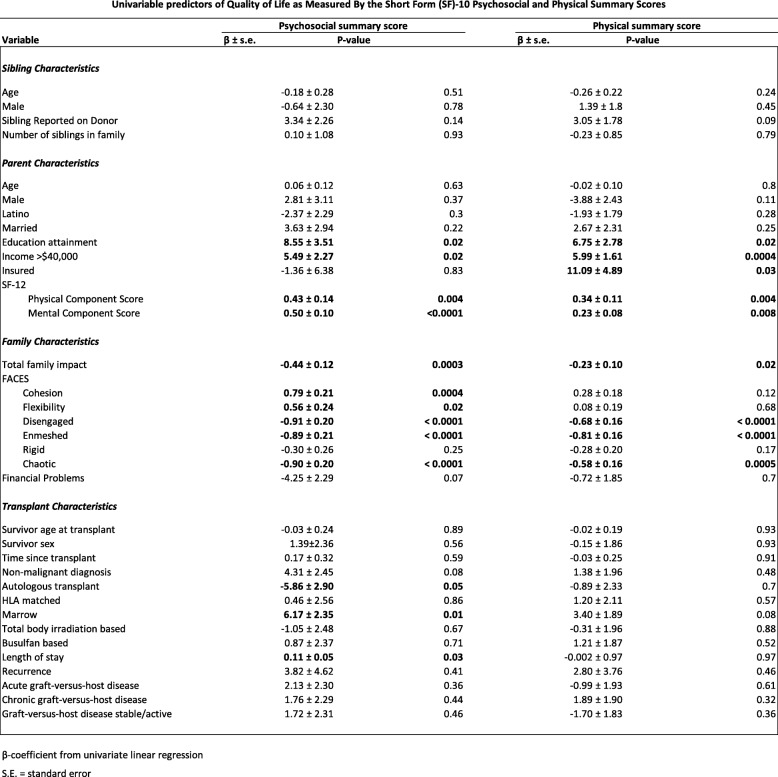
Univariable predictors of Quality of Life as Measured By the Short Form (SF)-10 Psychosocial and Physical Summary Scores

**Table 3:**
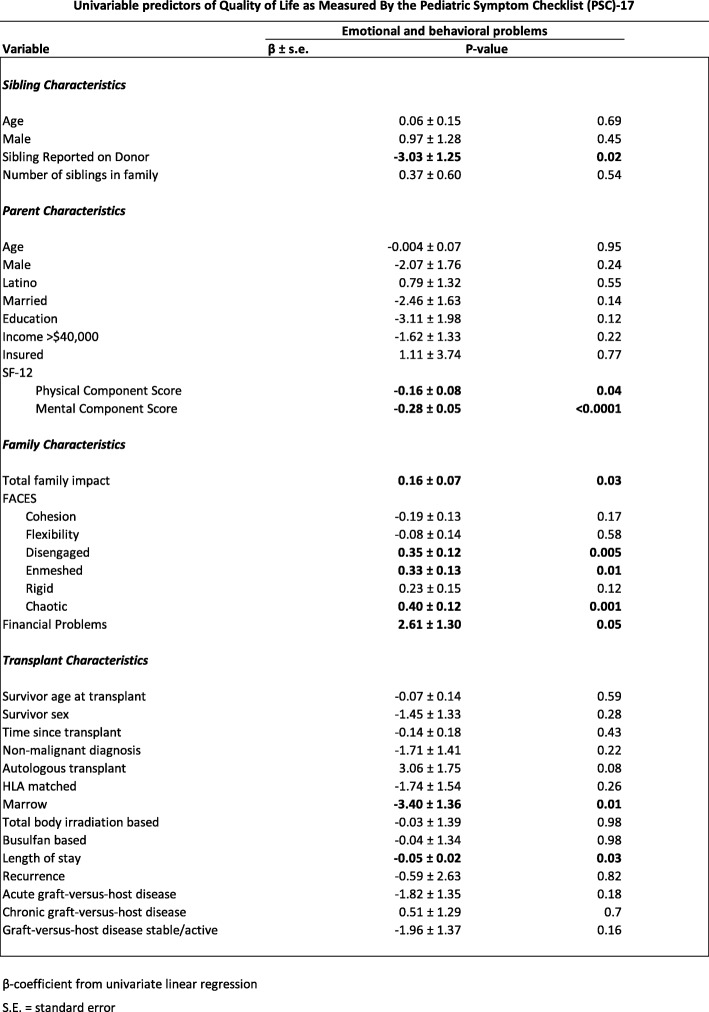
Univariable predictors of Quality of Life as Measured By the Pediatric Symptom Checklist (PSC)-17

**Table 4:** Multivariable Model - Predictors of Quality of Life as Measured By the Short Form (SF)-10 Physical Summary Scores

Variable	Physical summary score	
	β ± s.e.	*P*-value
Sibling Characteristics		
Sibling Reported on Donor	0.12 ± 1.88	0.95
Parent Characteristics		
SF-12		
Physical Component Score	0.15 ± 0.11	0.20
Mental Component Score	0.09 ± 0.09	0.35
Education attainment	1.63 ± 3.21	0.61
Income >$40,000	3.75 ± 1.94	0.06
Insured	7.86 ± 4.34	0.07
Family Characteristics		
Total family impact	− 0.08 ± 0.10	0.41
FACES		
Disengaged	0.08 ± 0.23	0.74
Enmeshed	− 0.28 ± 0.21	0.19
	− 0.18 ± 0.20	0.38
Transplant Characteristics		
Marrow	2.26 ± 1.85	0.23

β-coefficient from multivariable linear regression

*S.E.* Standard error

**Table 5:** Multivariable Model - Predictors of Quality of Life as Measured By the Short Form (SF)-10 Psychosocial Summary Scores

Variable	Psychosocial summary score	
	β ± s.e.	*P*-value
Parent Characteristics		
SF-12		
Physical Component Score	0.29 ± 0.15	0.06
Mental Component Score	**0.28 ± 0.12**	**0.03**
Education	3.23 ± 3.52	0.36
Income >$40,000	−5.13 ± 2.80	0.07
Family Characteristics		
Total family impact	0.07 ± 0.13	0.58
FACES		
Cohesion	**0.89 ± 0.37**	**0.02**
Flexibility	−0.03 ± 0.37	0.94
Disengaged	−0.45 ± 0.29	0.12
Enmeshed	−0.14 ± 0.29	0.63
Chaotic	−0.36 ± 0.25	0.16
Financial Problems	−2.00 ± 2.36	0.40
Transplant Characteristics		
Non-malignant diagnosis	0.26 ± 2.51	0.92
Autologous transplant	4.86 ± 4.30	0.26
Marrow	5.04 ± 3.11	0.11
Length of stay	0.04 ± 0.05	0.39

β-coefficient from univariate linear regression

*S.E.* Standard error

The boldface denotes a *p*-value < or equal to 0.05.

**Table 6:** Multivariable Model - Predictors of Quality of Life as Measured By the Pediatric Symptom Checklist (PSC)-17

Variable	Emotional and behavioral problems	
	β ± s.e.	*P*-value
Sibling Characteristics		
Sibling Reported on Donor	−1.27 ± 1.38	0.36
Parent Characteristics		
SF-12		
Physical Component Score	**−0.18 ± 0.08**	**0.03**
Mental Component Score	**−0.19 ± 0.07**	**0.01**
Family Characteristics		
Total family impact	−0.06 ± 0.08	0.47
FACES		
Disengaged	0.17 ± 0.16	0.31
Enmeshed	−0.05 ± 0.17	0.78
Chaotic	0.14 ± 0.15	0.38
Financial Problems	0.31 ± 1.29	0.81
Transplant Characteristics		
Autologous transplant	−3.22 ± 2.51	0.2
Marrow	−2.86 ± 1.86	0.13
Length of stay	−0.04 ± 0.02	0.07

β-coefficient from univariate linear regression

*S.E.* Standard error

The boldface denotes a *p*-value < or equal to 0.05.

## Discussion

Because siblings are relatively inaccessible given their relative absence in clinics and at the bedside, the assessment of QoL of siblings of HCT survivors is challenging. As a result, a robust appreciation of the impact of the HCT experience on families including siblings has remained relatively unexplored. To address this gap in knowledge, this study focused on the characterization of sibling QoL, defined as the health and well-being of the sibling, incorporating physical, mental, and social health [10, 11], as assessed by parent proxies. Consistent with the findings of others, we found that QoL mean subscale scores for the siblings did not differ significantly from norms. However, a subset of siblings appears to experience adverse psychosocial health and exceeded clinical thresholds for emotional and behavioral issues, as reported by parents. Moreover, we found that worse sibling QoL, as reported by parents, was associated with factors such as adverse parent QoL and family function.

On average parents reported that siblings of HCT survivors had overall good physical and psychosocial health. A greater understanding of which siblings can readjust their expectations surrounding the HCT experience in an adaptive fashion is needed as nearly 20% of siblings in our study had reduced psychosocial (> 1 SD below the normative mean) QoL, according to their parents. Despite overall good QoL, parents endorsed emotional and behavioral problems in their healthy siblings, as documented by PSC-17 total problem score elevation in 24% as compared to rates of 11% in the general population [23]. The identification of these siblings and their families, coupled with timely referral for care, is needed [34, 35]. Importantly, because imperfect agreement has been noted among sibling self-report and parent proxy-reporting, [36] identification of siblings at-risk for adverse QoL from parent report alone requires more complete assessment including self-report assessment and the use of other informants, when possible.

Parents’ reports of their own mental health are lower than norms. Given that an average of 5 years had elapsed since HCT, the results of diminished parental mental health are striking. Decrements in parent mental health in the context of survival after HCT may be due to a variety of ongoing stressors (e.g., frequent doctor visits, ongoing health issues, persistent financial burden). Our findings mirror other studies of parents in the setting of pediatric HCT, which document significant psychological distress during the 1st year post-HCT [17]. The identification of these parents that continue to experience ongoing distress is sobering. The presence of ongoing distress among parents also has important salience as a predictor of parent proxy reporting of sibling QoL. Previous assessments of QoL using parent proxy reporting of QoL have documented that siblings generally experience a greater burden of diminished QoL than is perceived by parents [36]. However, studies suggest that this may not be the case when considering the perceptions of distressed parents [36].

Following pediatric HCT, the family experiences ongoing disruptions in daily routines and daily life. This may support the ongoing sustenance of maladaptive patterns of family functioning. We found that family cohesion served as a predictor of parent proxy reports of sibling QoL, such that greater cohesion was associated with better sibling QoL. In cohesive families, parents may be able to more accurately depict the adaptation to the HCT experience. Moreover, parents in cohesive families may be more cognizant of alterations in mood or behaviors that are indicative of maladaptation. Other aspects of family functioning such as disengagement, enmeshment, or chaos were not associated with parent proxy reports of sibling QoL.

Among sociodemographic and HCT-related factors, only diminished parental income was found to be a salient factor associated with adverse sibling QoL, perceived by parents. Research studies have documented greater reports of psychosocial distress among families from lower socioeconomic backgrounds that may incur a greater financial burden in the context of chronic illness such as childhood cancer [37, 38]. Families with limited financial resources may have diminished instrumental and adaptive support resources to address the ongoing physical and psychological challenges of the HCT survivor experience. We did not identify any HCT-related factors that were significant predictors in our sample.

We acknowledge the study’s limitations. First, this study is a cross-sectional study, focusing on parent proxy responses with respect to the assessment of QoL among siblings of pediatric HCT survivors. Moreover, a control group was not included. Despite these limitations, it did allow for the characterization of parent proxy reports of sibling QoL in families impacted by HCT. It also allowed for the exploration of potential correlates of parent proxy reports of sibling QoL. We acknowledge that research demonstrates that concordance issues exist between child and parent proxy reports of QoL; however, a role for parent proxy reporting of sibling QoL exists. The ability to readily assess QoL of siblings of HCT survivors is challenged by their inaccessibility (i.e., they are not present in the clinic or at the bedside). While sibling self-report is preferred, particularly among older children, characterization of the sibling HCT experience is vital and underscores the need to characterize and understand the role of proxy reporting in the assessment of QoL among siblings of HCT survivors. Importantly, the sample represented was nearly 60% self-reported as Latino, which provides a unique strength to these analyses, as Latinos are frequently an underrepresented minority in the study of sibling QoL across all disease populations. However, because of the potential cultural differences in the perceptions of the family and its members, these results may not be generalizable to other racial and ethnic groups. Future longitudinal studies that utilize multiple informants including sibling self-report as well as appropriate control groups will be helpful.

## Conclusions

The main finding from this study of parent proxy reporting of QoL among siblings of HCT survivors is that siblings are a physically, mentally, and socially healthy group of individuals overall as perceived by parents. However, there are subsets of siblings at risk for developing adverse QoL including emotional and behavior problems as reported by parents. Parent QoL and specific aspects of family functioning (e.g., cohesion) are salient predictors of sibling QoL as reported by parents. Additional studies, longitudinal in design, incorporating multiple informants are needed to characterize and better understand the role of parent proxy reporting of sibling QoL and agreement with parent proxy reports and sibling self-reports in the setting of the HCT experience.

## Data Availability

The datasets used and analyzed during the current study are available from the corresponding author on reasonable request.

## Abbreviations

FACES IV: Family Adaptability and Cohesion Evaluation Scales, Version IV
GVHD: Graft-versus-host disease
HCT: Hematopoietic cell transplantation
IOF: Impact on Family
IRB: Institutional Review Board
MCS: Mental component summary
PCS: Physical component summary
PHS: Physical health summary
PSC-17: Pediatric Symptom Checklist − 17
PSS: Psychosocial health summary
PTSD: Post-traumatic stress disorder
QoL: Quality of life
SD: Standard deviation
SF: Short Form
TBI: Total body irradiation
US: United States
